# Association of Vitamin B6 and Homocysteine–ACTH Coupling With Depressive and Anxiety Symptoms in Burning Mouth Syndrome: An Exploratory Case–Control Study

**DOI:** 10.1111/joor.70253

**Published:** 2026-07-07

**Authors:** Ashley Lebel, Charlotte Guillouet, André Porporatti, Yves Boucher

**Affiliations:** ^1^ Department of Orofacial Pain Institute of Dental Surgery, Pitié‐Salpêtrière Hospital, Assistance Publique‐Hôpitaux de Paris (AP‐HP) Paris France; ^2^ Gene Regulation and Adaptive Behaviors, Center for Neuroscience Sorbonne University (NeuroSU) UMR CNRS 8265, Inserm 1341, Sorbonne Université Paris France; ^3^ Laboratory of Orofacial Neurobiology, Inserm UMR 1333 Oral Health, Université Paris Cité Montrouge France; ^4^ Department of Pediatric Dentistry Rothschild Hospital, Assistance Publique‐Hôpitaux de Paris (AP‐HP) Paris France

**Keywords:** biomarkers, burning mouth disorder, chronic pain, headache, hormones, nociplastic pain, oral dysaesthesia, orofacial pain, psychological distress, psychological stress

## Abstract

**Background:**

Burning mouth syndrome (BMS) is a chronic, idiopathic and debilitating orofacial pain disorder, increasingly discussed within oral dysaesthesia and nociplastic pain frameworks, in which psychological distress and stress‐related mechanisms are frequently implicated. Vitamin B6, homocysteine and adrenocorticotropic hormone (ACTH) reflect biologically interconnected micronutrient, one‐carbon‐metabolic and neuroendocrine pathways, but their relevance to BMS and particularly their interrelationships within patients remains unclear.

**Objectives:**

To compare plasma vitamin B6, homocysteine and ACTH concentrations between patients with BMS and controls, and to characterise the relationships among these biomarkers and with anxiety and depression within each group.

**Methods:**

In this exploratory case–control ancillary analysis of the OPIODYN study, plasma pyridoxal 5′‐phosphate (PLP; vitamin B6), homocysteine and ACTH were measured in 21 patients with BMS and 17 controls, and anxiety and depression were assessed with the Hospital Anxiety and Depression Scale (HADS). Between‐group comparisons used 2‐sided Mann–Whitney *U* tests with Hodges–Lehmann median differences and 95% confidence intervals (CIs). Within‐group Spearman correlations and between‐group differences in correlation strength (Δρ, Monte Carlo permutation) were computed, with Benjamini–Hochberg control of the false discovery rate within pre‐declared families.

**Results:**

No statistically significant between‐group differences were observed for PLP, homocysteine or ACTH concentrations. Median differences were +5.35 nmol/L (95% CI, −14.88 to 26.78; *p* = 0.567) for PLP, −0.13 μmol/L (95% CI, −1.81 to 1.49; *p* = 0.907) for homocysteine and −0.66 pmol/L (95% CI, −1.61 to 0.35; *p* = 0.186) for ACTH. Most participants in both groups had adequate PLP and within‐range homocysteine and basal ACTH. Homocysteine and ACTH were strongly correlated in patients with BMS (ρ = 0.806; 95% CI 0.562–0.891) but not in controls (ρ = 0.204; 95% CI, −0.344 to 0.668), with a significant between‐group difference in correlation strength (Δρ = 0.602; *p* = 0.018). Among patients with BMS, both HADS subscales showed positive correlations with homocysteine and ACTH (ρ = 0.61–0.64; all *q* = 0.010), whereas no corresponding correlation was significant in controls, and PLP was not correlated with HADS or with the other biomarkers in either group. No single biomarker discriminated BMS from control status (AUC, 0.51–0.63).

**Conclusion:**

These exploratory findings do not support vitamin B6, homocysteine, or ACTH as stand‐alone biomarkers of BMS. Rather, they suggest that psychological distress maps onto a homocysteine–ACTH correlation axis in patients with BMS, from which PLP appears dissociated, and support a shift in BMS biomarker research from isolated concentrations towards the relationships among metabolic, neuroendocrine and affective dimensions. Larger studies are needed to test the reproducibility of this pattern and its potential value for identifying clinically meaningful BMS subgroups.

## Graphical abstract

In this exploratory case–control study, plasma PLP (vitamin B6), homocysteine and ACTH concentrations did not differ between patients with burning mouth syndrome (BMS) and controls, and none discriminated patients individually. Within patients, however, psychological distress mapped onto a homocysteine–ACTH relationship from which PLP appeared dissociated. These findings support a shift in BMS biomarker research from isolated concentrations towards the relationships among metabolic, neuroendocrine and affective dimensions. Larger longitudinal studies are needed to test the reproducibility of this pattern, clarify its relationship to stress‐related pathophysiology, and determine whether it identifies clinically meaningful subgroups of patients with BMS.

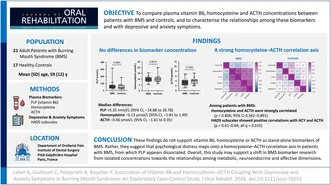

## 1 Introduction

Burning mouth syndrome (BMS) is a chronic idiopathic orofacial pain disorder defined by intraoral burning or dysaesthesia recurring on most days for more than three months, with clinically normal oral mucosa and no identifiable local or systemic cause [1, 2]. Its pathophysiology remains incompletely understood and is usually attributed to interacting neuropathic, endocrinological and psychosocial mechanisms, with limited and inconsistent treatment response and impaired quality of life [3, 4, 5, 6, 7, 8].

The definition and classification of BMS are still evolving, which complicates comparison across biomarker studies. Expert consensus has recommended renaming the condition to burning mouth disorder and revising its criteria [9], and research diagnostic criteria are still being formalised [10]. Recent work has also highlighted that current criteria may exclude patients with otherwise similar non‐burning intraoral dysaesthesia, and that diagnostic performance varies across classification systems [11, 12, 13]. BMS is accordingly being repositioned as the best‐defined phenotype within a wider spectrum of oral dysaesthetic and perceptual disorders (ODPD) under consideration for ICHD‐4 and ICOP‐2 [14]. Because the categorical definition is not yet settled, dimensions that remain stable across classifications, in particular the psychological and stress‐related features of BMS, are informative targets for biomarker investigation.

Anxiety and depression are the psychological characteristics most consistently associated with BMS and are elevated relative to controls across studies [15]. Stress‐related mechanisms have likewise been implicated [16], including higher cortisol and other stress markers at the group level [17]. Together, these observations support psychological distress and stress‐related physiology as clinically relevant dimensions of BMS. However, evidence remains limited as to whether biochemical markers linked to these dimensions relate to affective burden within patients.

Among the metabolic mechanisms proposed in BMS, micronutrient status has received sustained attention, mainly for metallic ions and the B‐group vitamins B1, B9 and B12 [18, 19]. Vitamin B6 has been comparatively overlooked despite biological plausibility, as both its deficiency and its excess are associated with peripheral neuropathy [20]. In patients with BMS, low vitamin B6 was historically proposed as a contributor to oral burning, but the evidence is inconsistent and methodologically heterogeneous. Early studies reporting low vitamin B status and symptomatic response to replacement used indirect assays and uncontrolled designs [21, 22]. Large‐scale screening has since identified low vitamin B6 in only a small minority of patients [23], whereas other observations suggest that a subgroup may instead show elevated concentrations [24, 25]. Vitamin B6 dysregulation in BMS may therefore extend beyond deficiency, which makes its biologically linked partners informative.

The active coenzyme form of vitamin B6, pyridoxal 5′‐phosphate (PLP), is a key cofactor in homocysteine metabolism, making homocysteine a biologically relevant companion marker [26]. Homocysteine has been linked to neuronal injury through oxidative and methylation‐dependent mechanisms [27, 28, 29], is higher in depression in meta‐analysis [30], and is elevated in a subset of patients with BMS [31]. Adrenocorticotropic hormone (ACTH), the pituitary hormone of the hypothalamic–pituitary–adrenal (HPA) axis, reflects neuroendocrine stress activity, a second pathway implicated in BMS [32, 33]. ACTH is also connected to vitamin B6 metabolism, because it increases hydrolysis of B6 vitamers in adrenal tissue [34], and PLP is in turn required for synthesis of γ‐aminobutyric acid and serotonin, two neurotransmitters that regulate the HPA axis [35, 36]. Homocysteine and ACTH therefore provide complementary markers of one‐carbon metabolism and neuroendocrine stress physiology that are individually relevant to BMS, but whose interrelationship within patients has not been characterised.

Most previous biomarker studies in BMS, including our own, have focused almost entirely on comparing concentrations between patients and controls, an approach that has produced null or inconsistent group‐level differences for most analytes [17, 32]. A smaller body of work has examined whether individual biomarkers, chiefly salivary cortisol, can serve as surrogates for psychological state in BMS, but this evidence is inconsistent and largely confined to single salivary stress and immune markers [37, 38, 39]. Whether circulating one‐carbon and neuroendocrine markers are related to one another, to anxiety and depression, and whether these relationships differ between patients with BMS and controls remains unknown. This exploratory study therefore had two aims. The first was to compare plasma PLP, homocysteine and ACTH concentrations between patients with BMS and controls. The second was to characterise within each group the correlations among the biomarkers and their associations with anxiety and depression, and to compare the strength of these relationships between groups, as a preliminary assessment of whether metabolic, neuroendocrine and affective measures are coupled in BMS.

## 2 Materials and Methods

### 2.1 Study Design and Objectives

This was an exploratory ancillary case–control analysis using baseline samples and data from the OPIODYN study, which investigated opiorphin in burning mouth syndrome (BMS) [40]. The OPIODYN study enrolled patients with BMS and matched healthy controls and was conducted at the Groupe Hospitalier Pitié‐Salpêtrière and the Institut Pasteur (Paris, France). Further details of the parent study have been reported in full [40].

The primary objective of the present study was to compare plasma concentrations of vitamin B6, measured as pyridoxal 5′‐phosphate (PLP), homocysteine and adrenocorticotropic hormone (ACTH) between patients with BMS and controls. Secondary objectives were to characterise, within each group, the correlations among these biomarkers and their associations with anxiety and depression, to compare the strength of these relationships between groups, and to quantify the discriminative value of each biomarker. This study followed the STrengthening the Reporting of OBservational studies in Epidemiology (STROBE) guidelines.

### 2.2 Participants

Patients with BMS and healthy controls were initially matched pairwise on age, sex and hormonal status, with 21 participants in each group at enrolment (two men and 19 women per group, of whom 14 women were postmenopausal and 6 were receiving hormone replacement therapy). No participant was excluded on diagnostic grounds in the present analysis. The parent cohort had already been screened for idiopathic BMS using the assessment described below, and the only attrition specific to this ancillary analysis was the exclusion of four controls for whom one or more biomarker measurements were missing. The final analytic sample comprised 21 patients with BMS and 17 controls. Because exclusion of these four controls broke the original matching structure, all biomarker analyses were conducted as unmatched case–control comparisons rather than matched‐pair analyses. Baseline characteristics, including age, body mass index (BMI), smoking habits, alcohol consumption, emotional state, pre‐sampling self‐reported stress, salivary measures and dental status, were assessed to evaluate comparability between groups (Table S1).

### 2.3 Diagnostic Assessment

Participants underwent standardised medical history taking, clinical examination and laboratory evaluation. Diagnosis and clinical assessment were performed by a senior examiner specialised in oral mucosa pathology. Idiopathic BMS was diagnosed using established IASP‐based criteria concordant with ICHD‐3 and ICOP [1, 2], namely oral burning or dysaesthesia recurring on most days for more than three months, clinically normal oral mucosa, and absence of an identifiable local or systemic cause.

The clinical examination assessed mucosal abnormalities, traumatic prostheses, hyposalivation and local conditions potentially associated with oral pain, including herpes, lichen planus, allergic stomatitis and mucositis. Screening for secondary causes also covered systemic diseases possibly associated with oral burning (including diabetes, anaemia, cerebrovascular disease, multiple sclerosis, malignancy, Sjögren syndrome, lupus and Lyme disease), nutritional deficiencies in B vitamins and iron, food and dental‐material allergies, gastro‐oesophageal reflux, use of medications known to be associated with oral burning or altered taste or sensation (e.g., angiotensin‐converting enzyme inhibitors, diuretics, anti‐vascular endothelial growth factor agents and neuroleptics) and other trigeminal pain disorders. When clinically indicated, blood tests for vitamins B9 and B12, ferritin, thyroid‐stimulating hormone and glucose and microbiological testing for *Candida albicans* and *Helicobacter pylori* , were performed. Allergy investigations were performed only when clinically suspected and consisted of standard cutaneous patch tests, with additional tests including metals (mercury, gold, palladium and platinum), acrylic resin components and selected oral hygiene products.

### 2.4 Inclusion and Exclusion Criteria

The BMS group included patients aged 18–80 years with oral pain of the tongue or oral mucosa, with or without xerostomia or dysgeusia, present for several hours per day for at least three months, with clinically normal oral mucosa and no systemic disorder known to be associated with oral burning sensations. Controls were healthy participants aged 18–80 years with no oral or dental pathology and no oral pain or discomfort. Participants were excluded for age younger than 18 years or older than 80 years, cognitive or communication impairment preventing completion of self‐reported ratings or questionnaires, concurrent participation in another biomedical research protocol, or absence of health insurance coverage.

### 2.5 Sample Collection and Biochemical Measurements

Blood samples were collected and processed under standardised fasting morning conditions between 9:00 and 12:00, as previously described [40]. To limit stress‐related variation, participants rested for 15 min before sampling in a quiet, temperature‐controlled room at 21°C. Blood samples were immediately placed in a peptide‐preservation cocktail, centrifuged within 2 h and stored as plasma in the dark at −80°C at the Institut Pasteur biobank until analysis. No temperature‐chain disruption occurred during storage.

PLP and total homocysteine were quantified by liquid chromatography–tandem mass spectrometry (LC–MS/MS), and ACTH was measured in EDTA plasma by electrochemiluminescence immunoassay (ECLIA) (CERBA, Paris, France). Concentrations were expressed in SI‐compatible molar units: PLP in nmol/L, homocysteine in μmol/L and ACTH in pmol/L. Laboratory reference intervals were 12–128 nmol/L for PLP, 5–15 μmol/L for homocysteine, and 2.3–10.6 pmol/L for ACTH.

Because assay‐specific laboratory reference intervals are not equivalent to nutritional adequacy thresholds, PLP was additionally interpreted using literature‐based thresholds, with concentrations below 20 nmol/L considered deficient, 20–30 nmol/L marginal and above 30 nmol/L adequate. This approach is consistent with recommendations identifying PLP as the most widely used and interpretable biomarker of vitamin B6 status and supporting the better interpretability of the 20 and 30 nmol/L thresholds compared with assay‐specific laboratory intervals alone [26, 41]. For homocysteine and ACTH, the assay‐specific laboratory reference intervals were broadly consistent with commonly used international reference intervals under similar preanalytical conditions.

### 2.6 Psychological, Stress and Salivary Measures

Anxiety and depression were assessed with the Hospital Anxiety and Depression Scale (HADS), which comprises separate seven‐item anxiety and depression subscales, each scored from 0 to 21 [42]. Perceived stress was recorded on a 0–100 mm visual analogue scale (VAS) immediately before blood sampling, and unstimulated whole salivary flow rate was measured. The VAS and salivary‐flow data were available only as group‐level summary statistics for the parent cohort, with 21 participants per group, and were therefore used descriptively. Because individual‐level data for these variables were not available in the ancillary dataset, they could not be included in correlation or regression analyses.

### 2.7 Statistical Analysis

Continuous variables were inspected graphically using histograms and density plots. Because biomarker distributions, particularly PLP, showed skewness with occasional extreme values, these values were retained and addressed with rank‐based methods rather than excluded. Biomarkers were summarised as medians with interquartile ranges (Q1–Q3) and compared between groups using 2‐sided Mann–Whitney *U* tests. Estimation was emphasised throughout, with group differences expressed as Hodges–Lehmann (HL) location shifts with 95% confidence intervals (CIs) and effect sizes expressed as the common‐language effect size (CLES), defined as the probability that a randomly selected BMS value exceeds a randomly selected control value, with ties counted as one half. Baseline continuous variables were summarised as means and standard deviations or as medians and interquartile ranges, and compared with Welch *t*‐test or Mann–Whitney *U* test, as appropriate. Categorical variables were summarised as counts and percentages and compared using Fisher exact or Fisher–Freeman–Halton exact tests.

Within‐group associations were quantified with Spearman rank correlations, reported as ρ with bootstrap percentile 95% CIs and 2‐sided *p* values. Between‐group differences in correlation strength were expressed as Δρ, defined as ρ in the BMS group minus ρ in the control group, with bootstrap percentile 95% CIs, and tested with 2‐sided Monte Carlo permutation tests that randomly reassigned group labels while preserving the observed group sizes. Associations of each biomarker with anxiety and with depression were examined within each group using the same approach.

To balance sensitivity against multiplicity, the primary comparisons were pre‐specified as the three between‐group biomarker differences and the homocysteine‐ACTH Δρ, and were interpreted using estimation with 95% CIs. Benjamini‐Hochberg control of the false discovery rate (FDR) was then applied within three pre‐declared families, comprising the six biomarker‐biomarker correlations, the 12 anxiety and depression to biomarker correlations and the three univariate logistic models. The between‐group Δρ comparisons were interpreted by estimation and were not included in the FDR families. Findings that did not survive FDR correction were interpreted as hypothesis‐generating. Descriptive and balance comparisons, including baseline characteristics, salivary flow and VAS stress, were not FDR‐corrected because their purpose was to characterise comparability rather than to test the study hypotheses.

Exploratory univariate logistic regression models were fitted with BMS status as the dependent variable and each biomarker entered separately as a continuous predictor. Odds ratios (ORs) were reported per 10 nmol/L for PLP, per 1 μmol/L for homocysteine and per 1 pmol/L for ACTH. No multivariable model was fitted, given the limited number of events and the risk of overfitting. Model fit was assessed using the likelihood‐ratio deviance test. Departure from linearity was evaluated using restricted cubic splines with three knots placed at the 10th, 50th and 90th percentiles, by comparing the spline model with the linear model. Discrimination was summarised with the area under the receiver‐operating‐characteristic curve (AUC) with bootstrap percentile 95% CIs, and McFadden pseudo‐*R* ^2^ and Tjur's *R* ^2^ were reported as descriptive indices of model fit.

As a sensitivity analysis, between‐group comparisons were repeated using Welch *t*‐tests, with consistent results and unchanged conclusions. Because this was an exploratory ancillary analysis rather than a biomarker study with a prespecified effect‐size target, no a priori sample‐size calculation was performed and no post hoc power was computed. Effect sizes and 95% CIs were therefore emphasised for precision. All resampling used a fixed random‐number seed to ensure reproducibility, with bootstrap CIs based on 10 000 resamples and permutation tests on 50 000 permutations. Analyses were performed using Stata/MP 17.0 (StataCorp LLC, College Station, TX, USA), and figures were generated using GraphPad Prism 10.2.1 (GraphPad Software, San Diego, CA, USA).

## 3 Results

### 3.1 Participant Characteristics

The analytic sample comprised 21 patients with BMS and 17 controls, after four controls were excluded for missing biomarker data. The groups were comparable in age (58.6 ± 11.7 vs. 58.9 ± 11.5 years), sex assigned at birth (90.5% women in both), menopausal status, body mass index, tobacco and alcohol use and dental status (Table S1). Anxiety and depression scores were higher in patients with BMS than in controls (HADS anxiety 10.0 ± 4.8 vs. 5.2 ± 3.1, *p* = 0.003; HADS depression 6.1 ± 4.5 vs. 1.9 ± 1.5, *p* = 0.001). Perceived stress immediately before sampling did not differ between groups (median 15 vs. 11; *p* = 0.35), nor did baseline unstimulated salivary flow (Table S1). No participant reported vitamin B6 supplementation at the time of sampling.

### 3.2 Biomarker Concentrations and Reference Range Distributions

Plasma vitamin B6 (pyridoxal 5′‐phosphate; PLP), homocysteine and ACTH are summarised in Table 1 and Figure 1. Between‐group differences were small and imprecisely estimated, with no statistically significant difference for any biomarker. Median PLP concentration was 52.36 (Q1–Q3, 40.46–74.38) nmol/L in the BMS group and 41.06 (37.49–75.57) nmol/L in controls, corresponding to a Hodges–Lehmann (HL) median difference of +5.35 nmol/L (95% CI, −14.88 to 26.78; *p* = 0.567; CLES, 0.556). Median homocysteine concentration was 9.69 (8.18–11.55) μmol/L in the BMS group and 10.04 (8.17–11.65) μmol/L in controls, corresponding to a HL median difference of −0.13 μmol/L (95% CI, −1.81 to 1.49; *p* = 0.907; CLES, 0.487). Median ACTH concentration was 3.35 (2.27–4.29) pmol/L in the BMS group and 4.05 (2.88–4.58) pmol/L in controls, corresponding to a HL median difference of −0.66 pmol/L (95% CI, −1.61 to 0.35; *p* = 0.186; CLES, 0.373). All three CLES values were close to 0.50, indicating little separation between groups.

**TABLE 1:** Biomarker concentrations and category distributions in patients with burning mouth syndrome and controls.

Biomarker	BMS (*n* = 21), median (Q1–Q3)	Controls (*n* = 17), median (Q1–Q3)	Group difference estimate (95% CI)	*p*	CLES
PLP (vitamin B6), nmol/L	52.36 (40.46–74.38)	41.06 (37.49–75.57)	+5.35 (−14.88 to 26.78)	0.567	0.556
Deficient (< 20 nmol/L)	0 (0.0%)	1 (5.9%)	—	0.186	—
Marginal (20–30 nmol/L)	1 (4.8%)	3 (17.6%)	—		—
Adequate (> 30 nmol/L)	20 (95.2%)	13 (76.5%)	—		—
Homocysteine, μmol/L	9.69 (8.18–11.55)	10.04 (8.17–11.65)	−0.13 (−1.81 to 1.49)	0.907	0.487
Below reference range	0 (0.0%)	0 (0.0%)	—	1.000	—
Within reference range	20 (95.2%)	16 (94.1%)	—		—
Above reference range	1 (4.8%)	1 (5.9%)	—		—
ACTH, pmol/L	3.35 (2.27–4.29)	4.05 (2.88–4.58)	−0.66 (−1.61 to 0.35)	0.186	0.373
Below reference range	5 (23.8%)	3 (17.6%)	—	0.705	—
Within reference range	16 (76.2%)	14 (82.4%)	—		—
Above reference range	0 (0.0%)	0 (0.0%)	—		—

*Note:* Continuous variables are presented as median (Q1–Q3), and category data as *n* (%). Between‐group comparisons of continuous variables were performed using the Mann–Whitney *U* test, with between‐group differences estimated using the Hodges–Lehmann median difference and 95% CIs. Effect size is reported as the common language effect size (CLES), defined as the probability that a randomly selected value in the burning mouth syndrome group is higher than a randomly selected value in the control group, with ties counted as 0.5. Category distributions were compared using the Fisher–Freeman–Halton exact test. For pyridoxal 5′‐phosphate (PLP), categories were defined using literature‐based thresholds: deficient (< 20 nmol/L), marginal (20–30 nmol/L) and adequate (> 30 nmol/L). For homocysteine and ACTH, categories were defined according to the CERBA laboratory reference intervals (5–15 μmol/L and 2.3–10.6 pmol/L, respectively). Although the CERBA laboratory reference interval for PLP was 12–128 nmol/L, PLP was categorised using literature‐based adequacy thresholds, as described in the Methods. Descriptive comparisons were not FDR‐corrected.

Abbreviations: ACTH, adrenocorticotropic hormone; BMS, burning mouth syndrome; CI, confidence interval; CLES, common‐language effect size; FDR, false discovery rate; PLP, pyridoxal 5′‐phosphate.

**FIGURE 1:**
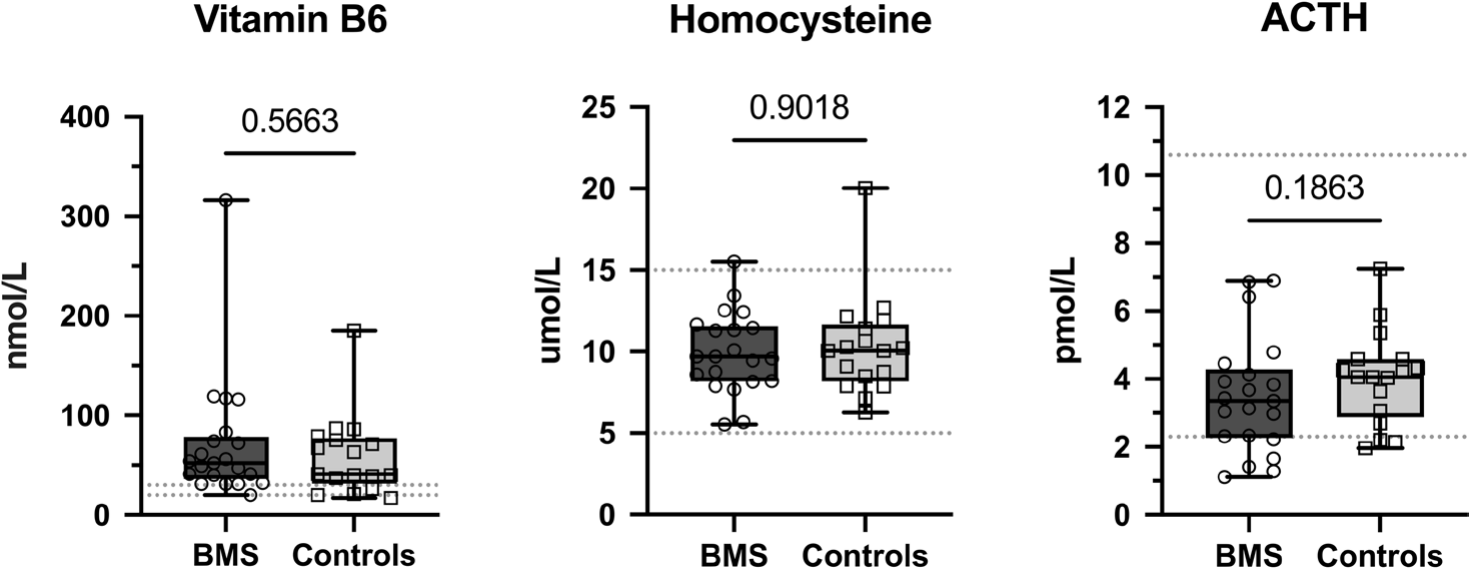
Vitamin B6, homocysteine and adrenocorticotropic hormone concentrations in patients with burning mouth syndrome and controls. Box‐and‐whisker plots show plasma concentrations of pyridoxal 5′‐phosphate (PLP; vitamin B6), homocysteine and adrenocorticotropic hormone (ACTH) in patients with burning mouth syndrome (BMS) and controls (CTRL). PLP is expressed in nmol/L, homocysteine in μmol/L, and ACTH in pmol/L. Boxes indicate the interquartile range (25th–75th percentiles), the horizontal line within each box indicates the median, whiskers extend to the most extreme data points within 1.5 times the interquartile range, and individual observations are superimposed. Dotted horizontal lines indicate interpretive thresholds or reference limits. For PLP, dotted lines mark the literature‐based adequacy thresholds used to classify vitamin B6 status as deficient (< 20 nmol/L), marginal (20–30 nmol/L) and adequate (> 30 nmol/L). For homocysteine and ACTH, dotted lines indicate the CERBA laboratory reference intervals (5–15 μmol/L and 2.3–10.6 pmol/L, respectively). Between‐group comparisons were performed using 2‐sided Mann–Whitney *U* tests, and exact *p* values are displayed above each panel. Corresponding numerical summaries are provided in Table 1.

Using prespecified literature‐based PLP thresholds, most participants in both groups were classified as adequate: 20 of 21 patients with BMS (95.2%) and 13 of 17 controls (76.5%); one patient with BMS (4.8%) and three controls (17.6%) were marginal, and one control (5.9%) was deficient (*p* = 0.186). Homocysteine was within the reference range in 20 of 21 patients with BMS (95.2%) and 16 of 17 controls (94.1%), with one above‐range value in each group (*p* = 1.000). ACTH was within range in 16 of 21 patients with BMS (76.2%) and 14 of 17 controls (82.4%), with below‐range values in five patients with BMS (23.8%) and three controls (17.6%), with no above‐range value in either group (*p* = 0.705) (Table 1). Using the assay‐specific laboratory interval for PLP, one participant in each group had a concentration above the upper reference limit of 128 nmol/L (317.73 nmol/L in the BMS group and 186.24 nmol/L in controls), whereas no PLP value was below the lower reference limit of 12 nmol/L.

### 3.3 Correlations Among Biomarkers

Within‐group correlations among biomarkers are reported in Table 2. Homocysteine and ACTH were strongly and positively correlated in patients with BMS (ρ = 0.806; 95% CI 0.562–0.891; *p* < 0.001) but not in controls (ρ = 0.204; 95% CI −0.344 to 0.668; *p* = 0.433), with a statistically significant between‐group difference in correlation strength (Δρ = 0.602; 95% CI 0.085–1.135; *p* = 0.018). This was the only biomarker‐biomarker correlation to remain significant after FDR correction within its family. PLP was not significantly correlated with homocysteine or ACTH in either group, and the between‐group difference was null for PLP and homocysteine (Δρ = −0.051; *p* = 0.86). The PLP–ACTH difference in correlation strength was numerically large but did not reach statistical significance and did not remain significant after FDR correction (Δρ = −0.612; *p* = 0.055) (Table 2).

**TABLE 2:** Within‐group Spearman correlations among biomarkers and between‐group differences in correlation strength in patients with burning mouth syndrome and controls.

Biomarker pair	BMS		Controls		Between‐group comparison	
	Spearman ρ (95% CI)	*p*	Spearman ρ (95% CI)	*p*	Δρ (95% CI)	Monte Carlo *p*
PLP (vitamin B6)—homocysteine	−0.232 (−0.581 to 0.197)	0.311	−0.181 (−0.657 to 0.394)	0.486	−0.051 (−0.720 to 0.628)	0.860
PLP (vitamin B6)—ACTH	−0.311 (−0.630 to 0.130)	0.170	+0.301 (−0.217 to 0.727)	0.241	−0.612 (−1.158 to 0.048)	0.055
Homocysteine—ACTH	0.806 (0.562–0.891)	< 0.001	0.204 (−0.344 to 0.668)	0.433	0.602 (0.085–1.135)	0.018

*Note:* Correlations were assessed using Spearman rank correlation coefficients (ρ) with bootstrap percentile 95% CIs and 2‐sided *p* values within each group. Between‐group comparisons are presented as the difference in Spearman correlation coefficients (Δρ = ρ BMS−ρ Controls) with bootstrap percentile 95% CIs and 2‐sided Monte Carlo permutation *p* values (50 000 permutations) based on random reassignment of group labels. Within the pre‐declared family of six biomarker–biomarker correlations, only the BMS homocysteine–ACTH correlation remained significant after Benjamini–Hochberg FDR correction.

Abbreviations: ACTH, adrenocorticotropic hormone; BMS, burning mouth syndrome; CI, confidence interval; FDR, false discovery rate; PLP, pyridoxal 5′‐phosphate.

### 3.4 Association of Depressive and Anxiety Symptoms With Biomarkers

Within patients with BMS, psychological distress, reflected by both HADS subscales, was positively correlated with homocysteine (anxiety ρ = 0.638; depression ρ = 0.609) and with ACTH (anxiety ρ = 0.613; depression ρ = 0.618), and all four correlations remained significant after FDR correction within the twelve‐member family (*q* = 0.010; Table 3, Figure 2). Neither anxiety nor depression was correlated with PLP in patients with BMS (ρ = −0.19 and −0.12), and none of the six corresponding correlations reached statistical significance in controls. Anxiety and depression were themselves strongly correlated in patients with BMS (ρ = 0.736) but only weakly correlated in controls (ρ = 0.244).

**TABLE 3:** Within‐group Spearman correlations of anxiety and depression with biomarkers in patients with burning mouth syndrome and controls.

Psychological distress and biomarker pair	BMS			Controls		
	Spearman ρ (95% CI)	*p*	*q* value	Spearman ρ (95% CI)	*p*	*q* value
Anxiety—Biomarker						
Anxiety—PLP (vitamin B6)	−0.186 (−0.532 to 0.203)	0.419	0.900	0.059 (−0.506 to 0.603)	0.823	0.900
Anxiety—Homocysteine	0.638 (0.304–0.854)	0.002	0.010^a^	−0.139 (−0.632 to 0.433)	0.596	0.900
Anxiety—ACTH	0.613 (0.260–0.809)	0.003	0.010^a^	−0.194 (−0.757 to 0.386)	0.456	0.900
Depression—Biomarker						
Depression—PLP (vitamin B6)	−0.115 (−0.512 to 0.298)	0.619	0.900	0.034 (−0.471 to 0.517)	0.897	0.900
Depression—Homocysteine	0.609 (0.240–0.843)	0.003	0.010^a^	0.097 (−0.415 to 0.583)	0.712	0.900
Depression—ACTH	0.618 (0.272–0.821)	0.003	0.010^a^	0.066 (−0.465 to 0.559)	0.801	0.900

*Note:* Correlations were assessed using Spearman rank correlation coefficients (ρ) with bootstrap percentile 95% CIs. The Benjamini–Hochberg procedure was applied across the pre‐declared family of 12 anxiety/depression–biomarker correlations, comprising six correlations in the BMS group and six in controls.

Abbreviations: ACTH, adrenocorticotropic hormone; BMS, burning mouth syndrome; FDR, false discovery rate; HADS, Hospital Anxiety and Depression Scale; PLP, pyridoxal 5′‐phosphate.

^a^ Remained significant after FDR correction. Anxiety and depression were measured with the Hospital Anxiety and Depression Scale and were themselves correlated within each group (ρ = 0.736 in BMS; ρ = 0.244 in controls).

**FIGURE 2:**
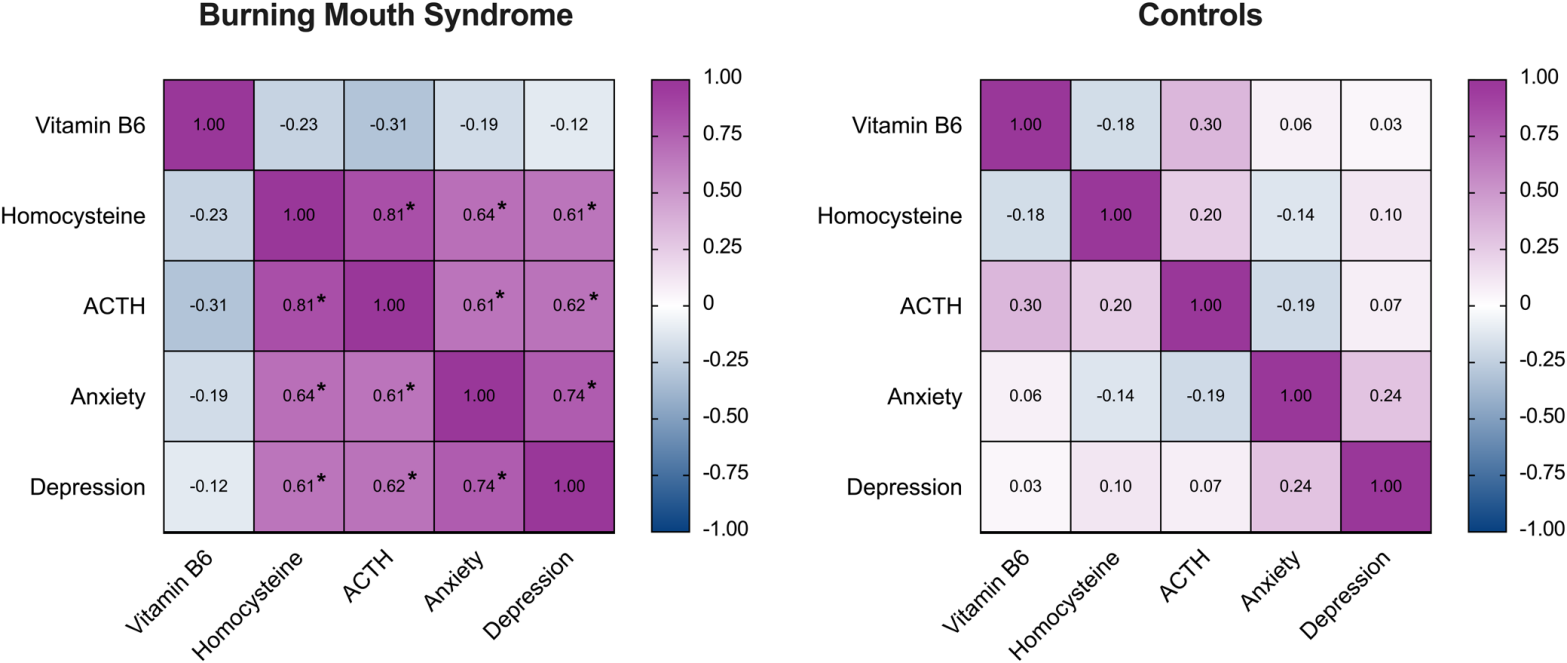
Within‐group correlation structure of biomarkers and psychological distress in burning mouth syndrome and controls. Heatmaps show pairwise Spearman correlation coefficients among PLP (vitamin B6), homocysteine, ACTH, HADS anxiety and HADS depression scores, shown separately for patients with BMS and controls. In patients with BMS, homocysteine and ACTH were strongly positively correlated, and both were positively correlated with HADS anxiety and depression scores; PLP showed no corresponding correlation with the other biomarkers or with HADS scores. No corresponding correlation pattern was observed in controls. Asterisks mark correlations that remained significant after Benjamini–Hochberg FDR correction within their pre‐declared family (*q* ≤ 0.018). Colour encodes the magnitude and sign of ρ; coefficients, 95% CIs and *q* values are given in Tables 2 and 3.

### 3.5 Discriminative Performance of Individual Biomarkers

Exploratory univariate logistic regression analyses did not identify a statistically significant association between any biomarker and BMS status (Table S2). Odds ratios were close to one for PLP (OR, 1.05 per 10 nmol/L; 95% CI 0.91–1.20; *p* = 0.50), homocysteine (OR, 0.95 per μmol/L; 95% CI 0.75–1.21; *p* = 0.70) and ACTH (OR, 0.80 per pmol/L; 95% CI 0.52–1.22; *p* = 0.30). Discriminative performance was limited, with AUCs of 0.556 (95% CI, 0.422–0.753) for PLP, 0.513 (95% CI, 0.450 to 0.717) for homocysteine and 0.627 (95% CI, 0.476–0.804) for ACTH. Likelihood‐ratio deviance tests and spline‐based assessments of nonlinearity were not statistically significant for any biomarker. McFadden pseudo‐*R* ^2^ and Tjur's *R* ^2^ values were uniformly low, indicating minimal incremental model fit and poor separation of predicted probabilities when biomarkers were considered individually, and no model remained significant after FDR correction (Table S2).

## 4 Discussion

### 4.1 Main Findings

In this exploratory case–control study, plasma vitamin B6 (PLP), homocysteine and ACTH did not differ between patients with BMS and controls, and no single biomarker discriminated patients individually. The groups differed less in biomarker concentrations than in how these biomarkers related to one another and to psychological distress. Homocysteine and ACTH were strongly correlated in patients but not in controls, and within patients both were associated with anxiety and depression, whereas PLP was unrelated to the other biomarkers or to affective symptoms in either group. Thus, in this cohort, patients with BMS did not show higher circulating concentrations of PLP, homocysteine or ACTH, although smaller concentration differences cannot be excluded. Rather, psychological distress mapped onto a homocysteine–ACTH relationship from which PLP appeared dissociated.

### 4.2 Homocysteine–ACTH Coupling and Stress‐Related Physiology

We have previously proposed that stress may have a pivotal role in the onset and exacerbation of BMS, in a two‐step model in which a stressor acting on a susceptible individual promotes central sensitisation [16]. Although the present findings do not test that model directly, they do identify a plausible biological interface between psychological distress, one‐carbon metabolism and HPA‐axis activity. Activation of the HPA‐axis accompanies depression and chronic stress, with meta‐analyses reporting raised cortisol and, to a lesser extent, raised ACTH [43]. Several lines of evidence also link cortisol and glucocorticoid exposure to homocysteine. Patients with active Cushing's syndrome, a state of endogenous cortisol excess, have higher homocysteine than patients in remission or controls [44], and corticosteroid therapy can raise homocysteine [45], although short‐term experimental administration of ACTH or cortisol has not consistently raised it. Glucocorticoids released on HPA‐axis activation induce cellular oxidative stress [46], and homocysteine both contributes to oxidative stress and reflects impaired methylation [27, 28, 29], while ACTH increases the turnover of B6 vitamers in adrenal tissue in an animal model [34]. A comparable interrelationship between homocysteine, cortisol and psychological state has also been described in type 2 diabetes [47].

This evidence makes the homocysteine–ACTH correlation observed in patients with BMS biologically plausible, although it should be interpreted as an exploratory association rather than evidence of a causal pathway. The wider spread of ACTH values in patients may have contributed to the correlation, and limited precision in the control group cannot be excluded. However, the absence of a comparable correlation in controls was not readily explained by restricted homocysteine variability, because homocysteine values were at least as variable in controls as in patients rather than confined to a narrow range that could obscure an association.

We did not reproduce the higher homocysteine reported in the much larger series of Lin et al. (2013), in which mean homocysteine was higher in patients than controls (10.9 vs. 8.6 μmol/L) and a value above the control mean plus two standard deviations was present in 22.3% of patients versus 4.8% of controls [31]. Their patient distribution was markedly right‐skewed, extending to 133.1 μmol/L, whereas ours lay largely within the conventional 5–15 μmol/L range. With 399 patients, that study was far better powered than ours to detect a difference in central tendency, so the discrepancy likely reflects both their greater power and their right‐skewed distribution, as well as the use of a study‐specific threshold rather than a conventional clinical reference range. In our data, homocysteine and ACTH covaried within patients despite concentrations comparable to controls, suggesting altered coordination rather than elevation of either biomarker.

### 4.3 Psychological Distress and Biomarker Interrelationships

Among patients with BMS, anxiety and depression were each associated with homocysteine and with ACTH. These associations are consistent with prior work in BMS, in which salivary cortisol is the most consistently reported stress‐related marker [39] and has been related to anxiety [37], depressive symptoms have been associated with cortisol within patients [32], and self‐perceived stress has been associated with higher cortisol and a higher cortisol‐to‐ACTH ratio [33]. The homocysteine findings are also consistent with evidence from non‐BMS populations, including meta‐analytic evidence linking homocysteine with affective symptoms [30], longitudinal population data linking higher homocysteine concentrations and high homocysteine trajectories to depressive symptom burden with heterogeneity by anxiety level and sociodemographic context [48] and nitrous‐oxide‐related neurological disease, where higher homocysteine accompanies greater anxiety and depression [49]. However, anxiety and depression were strongly intercorrelated in patients, so the four correlations should not be interpreted as four independent psychological‐biomarker findings. Rather, they point to two linked relationships: psychological distress with homocysteine and psychological distress with ACTH. The analyses were exploratory and correlational, and the relationships are best regarded as hypothesis‐generating.

### 4.4 Dissociation of Vitamin B6 From the Homocysteine–ACTH Coupling

PLP need not track homocysteine, because it is the cofactor for only one arm of homocysteine metabolism, the transsulphuration pathway, whereas remethylation depends on folate and vitamin B12 [26, 28]. Circulating PLP also reflects dietary intake and turnover [26] and is sensitive to HPA‐axis activity in animal tissue [34]. Its lack of association with homocysteine, ACTH, or affective symptoms is therefore metabolically plausible rather than paradoxical. Vitamin B6 has in addition rarely been measured in pain disorders: a migraine meta‐analysis found no study reporting serum pyridoxine, despite consistently raised homocysteine in that condition [50].

Our data argue against vitamin B6 deficiency as a feature of BMS, since no patient with BMS was deficient and the only deficient value in the sample came from a control. They also argue against a disease‐specific excess, since the two isolated above‐range values, one in each group and neither with reported supplementation, are difficult to interpret as specific to the disorder. Lamey et al. (1986) reported deficiencies of thiamine, riboflavine, or vitamin B6 in some patients, but assessed these vitamins indirectly, from the activation of red‐cell enzymes by added vitamins rather than from plasma PLP, and reported symptom resolution after uncontrolled replacement [21]. Hugoson and Thorstensson (1991), in a placebo‐controlled study, identified low thiamine and riboflavine rather than vitamin B6 and observed no benefit from replacement [22]. A contemporary Mayo Clinic screening series found low vitamin B6 in only 5.7% of patients [23]. The apparent frequency of vitamin B6 abnormality in BMS therefore depends heavily on the circulating form measured and the threshold applied. Our plasma‐PLP findings align more closely with contemporary screening data than with older reports suggesting frequent deficiency.

### 4.5 Concentrations, Discrimination and Disease Specificity

That the groups did not differ on any biomarker, and that none discriminated cases from controls, is consistent with prior evidence. The ACTH effect reported in depression is small [43], and a recent meta‐analysis in BMS points to raised cortisol rather than ACTH [17]. Because cortisol lies one step downstream of ACTH, a modest cortisol signal need not be accompanied by a basal ACTH difference large enough to detect. None of the three analytes separated patients from controls, with areas under the curve close to chance, which is consistent with the broader difficulty of identifying biomarkers specific to BMS. No current classification system distinguishes the disorder with both high sensitivity and high specificity [13], salivary stress markers show limited disease specificity [39], and BMS is increasingly discussed as a nociplastic pain condition involving altered nociceptive processing and central sensitisation [51] and as one phenotype within a wider spectrum of oral dysaesthesia [14, 52].

This distinction between within‐patient associations and disease classification has been observed in previous BMS studies. Biological measures may relate to symptom dimensions without functioning as disease‐specific classifiers. An earlier immunoendocrine study found that immune rather than HPA‐axis measures distinguished patients with BMS [32], and a recent network analysis found that multiple biological and psychological factors were embedded in associative pathways around BMS, with cortisol mediating several of these relationships and only nonspecific central‐sensitisation symptoms showing notable predictive capacity for BMS [53]. The present results extend this logic to circulating PLP, homocysteine and ACTH. Considered in isolation, these biomarkers were not diagnostically informative; considered together with psychological distress, they revealed a pattern that group‐level comparisons alone would have missed.

### 4.6 Limitations

Several limitations should be considered. The sample was small and was not powered to detect small or moderate group differences or to support subgroup analyses, and no post hoc power was computed. Correlation estimates in samples of this size are also sensitive to influential observations, although rank‐based methods reduce the impact of extreme raw values. The cross‐sectional design precludes causal and temporal inference, and unmeasured confounding cannot be excluded. The neuroendocrine assessment rested on a single basal ACTH measurement without cortisol or dynamic testing, and because ACTH secretion is pulsatile and circadian [54], a single value cannot capture dynamic HPA‐axis function. Perceived stress immediately before sampling was comparable between groups, which argues against differential acute stress as an explanation for the similar ACTH concentrations but does not address dynamic differences in stress responsivity. Plasma concentrations may not reflect the tissue or intracellular compartments where these markers act. Unstimulated salivary flow was low‐normal in both groups and did not differ, which argues against hyposalivation as a major confounder [13, 55], although salivary flow and perceived‐stress scores were available only as group summaries. Consequently, these variables could not be examined as individual‐level correlates of the biomarker findings or included in adjusted models. The anxiety and depression correlations, although they remained significant after FDR correction within their pre‐declared family, are exploratory and correlational.

### 4.7 Conclusion

In this exploratory case–control study, plasma PLP, homocysteine and ACTH concentrations did not differ between patients with BMS and controls, and none discriminated patients individually. Within patients, however, psychological distress mapped onto a homocysteine–ACTH relationship from which PLP appeared dissociated. These findings support a shift in BMS biomarker research from isolated concentrations towards the relationships among metabolic, neuroendocrine and affective dimensions. Larger longitudinal studies are needed to test the reproducibility of this pattern, clarify its relationship to stress‐related pathophysiology, and determine whether it identifies clinically meaningful subgroups of patients with BMS.

## Author Contributions

**André Porporatti:** validation, writing – review and editing. **Ashley Lebel:** writing – review and editing, writing – original draft, methodology, data curation, investigation, validation, formal analysis, supervision, visualisation. **Charlotte Guillouet:** methodology, validation, formal analysis, visualisation, writing – review and editing. **Yves Boucher:** conceptualization, methodology, data curation, investigation, validation, supervision, funding acquisition, project administration, resources, writing – review and editing.

## Funding

This research was supported by Assistance Publique‐Hôpitaux de Paris (#P081106).

## Ethics Statement

The study complied with the Declaration of Helsinki and Good Clinical Practice. Ethical approval for the study was given by the Institutional Review Board Ile de France VI (CPP#77–09—IDRCB: 2009‐A0096255), and the study was registered under #NCT02686359 at ClinicalTrials.gov. Further authorisation for the ancillary study was obtained in November 2024 (#20241122104145).

## Consent

All subjects provided written informed consent before inclusion, and all data were handled in accordance with participant confidentiality requirements.

## Conflicts of Interest

The authors declare no conflicts of interest.

## Supporting information


**Table S1:** Baseline demographic, clinical and dental characteristics, including salivary‐flow and perceived‐stress measures, in the original matched sample of patients with burning mouth syndrome and controls.

**Table S2:** Exploratory univariate logistic regression analyses and discriminative performance of plasma biomarkers for burning mouth syndrome status.

## Data Availability

The data that support this study's findings are available from AP‐HP, but restrictions apply. These data were used under licence for the current research and are not publicly available. However, data are available from the authors upon reasonable request and with permission of the AP‐HP institutional review board and Sorbonne University ethics committee.
