# Supplementary material for: Association of Vitamin B6 and Homocysteine–ACTH Coupling With Depressive and Anxiety Symptoms in Burning Mouth Syndrome: An Exploratory Case–Control Study
Source: J Oral Rehabil. 2026 Jul 7;53(11):2053–63. doi: 10.1111/joor.70253 (PMC13653300; doi:10.1111/joor.70253)
Supplement: Supplementary file 1 [file JOOR-53-2053-s001.docx]

Supplementary Table S2. Exploratory univariate logistic regression analyses and discriminative performance of plasma biomarkers for burning mouth syndrome status.

| Biomarker | OR scaling | OR  (95% CI) | Wald P value | Likelihood-ratio deviance test, χ² | Deviance test P value | Deviation from linearity P value | AUC  (95% CI) | McFadden pseudo-R² | Tjur’s R² |
| --- | --- | --- | --- | --- | --- | --- | --- | --- | --- |
| PLP (vitamin B6) | Per 10 nmol/L increase | 1.05  (0.91 to 1.20) | 0.503 | 0.505 | 0.477 | 0.897 | 0.556  (0.422 to 0.753) | 0.009 | 0.011 |
| Homocysteine | Per 1 µmol/L increase | 0.95  (0.75 to 1.21) | 0.700 | 0.149 | 0.699 | 0.972 | 0.513  (0.450 to 0.717) | 0.003 | 0.004 |
| ACTH | Per 1 pmol/L increase | 0.80  (0.52 to 1.22) | 0.300 | 1.115 | 0.291 | 0.176 | 0.627  (0.476 to 0.804) | 0.021 | 0.029 |

Note. Univariate logistic regression models were fitted separately for each biomarker, with burning mouth syndrome (BMS) status as the dependent variable. To improve interpretability and comparability across biomarkers, odds ratios (ORs) are reported per 10 nmol/L increase for pyridoxal 5′-phosphate (PLP; vitamin B6), per 1 µmol/L increase for homocysteine, and per 1 pmol/L increase for adrenocorticotropic hormone (ACTH). The overall deviance test corresponds to the likelihood-ratio comparison between the biomarker model and the intercept-only model. Potential deviation from linearity was assessed using restricted cubic splines with 3 knots placed at the 10th, 50th, and 90th percentiles, by comparing the spline model with the linear model; a non-significant result provided no evidence against the linear specification. Discriminative performance is summarised by the area under the receiver operating characteristic curve (AUC) with bootstrap percentile 95% CIs based on 10,000 resamples. McFadden pseudo-R² and Tjur’s R² are additionally reported as descriptive indices of model fit and discrimination. All analyses were exploratory; tests were 2-sided, and no model remained significant after Benjamini–Hochberg FDR correction. ACTH, adrenocorticotropic hormone; AUC, area under the receiver-operating-characteristic curve; BMS, burning mouth syndrome; CI, confidence interval; FDR, false discovery rate; OR, odds ratio; PLP, pyridoxal 5′-phosphate.
