# Supplementary material for: Association of Vitamin B6 and Homocysteine–ACTH Coupling With Depressive and Anxiety Symptoms in Burning Mouth Syndrome: An Exploratory Case–Control Study
Source: J Oral Rehabil. 2026 Jul 7;53(11):2053–63. doi: 10.1111/joor.70253 (PMC13653300; doi:10.1111/joor.70253)
Supplement: Supplementary file 2 [file JOOR-53-2053-s002.docx]

**Supplementary Table S1.** Baseline demographic, clinical and dental characteristics, including salivary-flow and perceived-stress measures, in the original matched sample of patients with burning mouth syndrome and controls.

| **Characteristic** | **BMS (n = 21)** | **Controls (n = 21)** | **P value** |
| --- | --- | --- | --- |
| Age, mean ± SD, y | 58.55 ± 11.68 | 58.93 ± 11.51 | 0.916 |
| Female sex assigned at birth, n/N (%) | 19/21 (90.5) | 19/21 (90.5) | 1.000 |
| Postmenopausal, n/N (%) | 14/19 (73.7) | 14/19 (73.7) | 1.000 |
| BMI, mean ± SD, kg/m² | 21.88 ± 2.99 | 23.99 ± 4.94 | 0.104 |
| HADS anxiety score, mean ± SD | 10.03 ± 4.75 | 5.24 ± 3.08 | 0.003 |
| HADS depression score, mean ± SD | 6.05 ± 4.48 | 1.85 ± 1.53 | 0.001 |
| Tobacco use, n/N (%) | 0/21 (0.0) | 3/21 (14.3) | 0.081 |
| Alcohol use, n/N (%) | 12/21 (57.1) | 14/21 (66.7) | 0.161 |
| Vitamin B6 supplement use, n/N (%) | 0/21 (0.0) | 0/21 (0.0) | 1.000 |
| Unstimulated salivary flow, mean ± SD, mL/min | 0.26 ± 0.16 | 0.30 ± 0.36 | 0.673 |
| Stress before sampling, median (Q1–Q3), 0–100 VAS | 15 (6–30) | 11 (3–18) | 0.350 |
| Dental characteristics |  |  |  |
| Number of missing teeth, mean ± SD | 4.55 ± 3.63 | 5.94 ± 3.58 | 0.219 |
| Number of root canal–treated teeth, mean ± SD | 3.70 ± 2.93 | 4.21 ± 2.99 | 0.580 |

**Note.** Continuous variables are presented as mean ± SD unless otherwise indicated, and categorical variables as n/N (%). Between-group comparisons were performed using Welch t tests or Mann–Whitney U tests for continuous variables and Fisher exact tests for categorical variables. Menopausal status was reported among women only. Salivary flow and VAS self-reported stress, available as group-level summary statistics, are reported descriptively. These descriptive variables were available for the original matched sample; the final biomarker analyses included 17 controls with complete biomarker data. All tests were 2-sided, and exact P values are reported. BMI, body mass index; BMS, burning mouth syndrome; HADS, Hospital Anxiety and Depression Scale; VAS, visual analogue scale.
